# Generated heat at implant site during gradual and single-drill protocols among Indian patients

**DOI:** 10.6026/97320630019881

**Published:** 2023-08-31

**Authors:** Shivendra Choudhary, Leena Priya, Taruna, Kumari Upasana, Abhishek Sinha, Deep Sundar

**Affiliations:** Department of Dentistry, Patna Medical College, Patna-4, Bihar, India; MDS Department of Dentistry, Patna Medical College and Hospital, Patna - 4, Bihar, India; Department of Dentistry, Patna Medical College and Hospital, Patna -4, Bihar, India; Department of Dentistry, Sri Krishna Medical College and Hospital, Muzaffarpur, Bihar, India; Department of Dentistry, Patna medical College, Bihar, India; Department of Public Health Dentistry, Buddha Institute of Dental Sciences and Hospital, Patna, Bihar, India; , Rajasthan, India

**Keywords:** Alveolar bone, bone blocks, dental implants, implant drill, osteotomy

## Abstract

Excessive heat generation during bone drilling for dental implant placement is a known risk factor for bone necrosis and delayed healing. Therefore, it is of interest to evaluate the maximum change in temperature during and after preparation of the implant site for an implant diameter of 4.2 using gradual drilling and single drilling protocols. Hence, 26 artificial bone blocks with d1 density were divided into two groups where the group I had 13 sites prepared using a single drill and for group II bone blocks, 13 implant sites were prepared with the gradual drill protocol using 5 drills. The drill was done at room temperature with 1500 rpm using constant saline irrigation of 50ml/min. The maximum change in temperature was assessed using an intraoral camera. The data collected were statistically evaluated and results were formulated. Data shows that temperature change was significantly higher in group II where a gradual drill protocol was done compared to group I with a single drill protocol for placing the dental implant of diameter 4.2.Considering its limitations, the present in-vitro assessment concludes that a single drill protocol for preparing an osteotomy site for placing a dental implant of diameter 4.2 generates lesser heat than conventional gradual drilling protocols.

## Background:

The majority of the workers considered a delicate, atraumatic, and direct surgical procedure as predictive criteria for long-term dental implant success as mechanical or thermal harm during implant site preparation can lead to bone necrosis and failure of osseointegration. [1] These factors include bone structure, surgical technique, drill geometry, irrigation factors, drill conditions, manufacturing materials, sharpness, osteotomy time, osteotomy load, and drilling turning speed. [2] Various previous research and studies have focused on the different drilling techniques and the temperature increase during and following drill, and its effect on the alveolar bone. [3] The conventional drilling method is based on implant site preparation with a set of drills where with each consecutive drill; the diameter of the osteotomy site is increased gradually by removing a small amount of alveolar bone. [4] Very few studies in the literature have compared gradual osteotomy preparation to single drilling to find the accurate technique related to heat generation in the alveolar bone. However, the data reported is scarce and inconclusive. [5] Different in-vitro studies have used human ex vivo bone, polyurethane foam synthetic bone models, and bovine bone ribs [6]. However, in the human mandible and maxilla, bones bone densities differ from D1 to D4 which questions the conducted studies and points to reconsideration of the study design as different artificial bone blocks of varying densities are available. [7] Therefore, it is of interest to evaluate the maximum change in temperature during and after preparation of the implant site for an implant diameter of 4.2 using gradual drilling and single drilling protocols.

## Materials and Methods:

The present study was done at Department of Dentistry, Patna Medical College, Patna 4, and Bihar, India. The ethical committee clearance was not needed as it was an in-vitro study.

## Study design:

The study included 26 samples and sites which were divided into two groups where the group I, 13 sites for implants were prepared using a single drill of 4 mm, and in Group II, 13 sites were prepared using conventional 5 drills of diameter 2, 2.8, 3.0, 3.5, and 4 mm.

## Methodology:

The study included artificial bone blocks of density D1. The bone blocks utilized in the present study were made of SRPF (solid rigid polyurethane foam) which is based on bone density made of D1 type of 0.48g/cm3. For the two groups, a drilling depth of 12mm was set. The drill speed was kept at 1500rpm and done at the room temperature of 25±1°C with constant irrigation using normal saline at the rate of 50ml/min. For each group, a new set of drills was used. For the present in-vitro assessment, special brackets were formulated that held the sample stationary during the whole drilling process and osteotomy site preparation. Also, these brackets helped in isolation during irrigation so the temperature can be controlled by preventing interference of readings from the infrared camera which could otherwise lead to errors in measurement.

Initially, before the drilling was started, the temperature was kept similar to the room temperature within the range of 23.0 to 290C. Each of the drills was placed on polyurethane foam blocks in a manner that after the drill was complete; the remaining wall thickness was between 0.3-0.7mm to get osteotomy in the area where it is easier for the infrared camera to assess temperature alterations. This was adapted in the present study to decrease bias affecting the change in the temperature. The infrared camera was placed at a distance of 50 cm from the study samples to attain the maximum spatial resolution. During and following the drilling process, thermal videos were recorded with the infrared intraoral camera. The camera placement was identical for each parameter where it was placed at a distance of 50 cm from the bone block with 95% emissivity, 50% of relative humidity, and a temperature of 25 ± 3°C.

## Statistical Analysis:

The data gathered was subjected to statistical analysis by incorporating SPSS (Statistical Package for the Social Sciences) Version 22.0; Chicago, IL, USA software using a student's t-test with 95% CI (confidence interval) to assess the change in the temperature between the two groups.

## Results:

It is of interest to evaluate the maximum change in temperature during and after preparation of the implant site for an implant diameter of 4.2 using gradual drilling and single drilling protocols. On assessing the initial temperature before drilling (T0) to maximum temperature after drilling (T max) for both conventional and single drilling, the results are summarized in Table 1. In conventional drilling the maximum initial temperature, T0 was seen with the drill of 2.0 mm where the maximum temperature recorded before drilling was 28.30 for the bone blocks. After drilling, the maximum temperature recorded for the conventional drilling was reported to be 44.80 which was a temperature high enough to damage the alveolar bone. In the bone block where the post-drill maximum temperature was 44.80the temperature before drilling was 26.1°. For the single drill group, a group I, the maximum temperature before drilling, T0 was 28.30 which were similar to group II. However, the T max for group I was 33.90 which was significantly lesser than group II, where it was 44.80 as shown in Table 1.

The change in temperature in the present study was denoted by ΔT, in the Group II where conventional drilling was done, with the 2.0mm drill, the maximum temperature change difference was found to be 6.4°C, with the 2.8 mm drill the ΔT highest was seen as 18.8°C, for 3.0mm drill it was 6.2°C, with 3.5mm drill was 8.7°C, and for the drill of 4.0mm, it was reported to be 9.1°C. For the group I bone blocks where a single drill was used, the temperature change was reported to be a maximum of 11.4°C. This temperature change in group I was 11.4°C, which was lesser than compared to group II where it was 18.8°C as depicted in Table 2.

For group I, single drill, the mean temperature change was 5.15±3.45 with a maximum temperature change limit of 1.3 and ΔT min limit was 11.4. This was significantly higher with group II where a conventional drill was used. The mean temperature change was 9.89±3.64, the ΔT max limit was 3.8 and the ΔT min limit was 18.5. This difference was significantly higher with conventional drills compared to single drills showing more heat generation with multiple conventional drills as shown in Table 3.

On assessing the statistical significance between the temperatures change in Group I for Group II where multiple conventional rills were used for osteotomy site preparation to place the dental implants. The ΔT, the mean difference was found to be -4.72, the t-value was -3.254, and the p-value was 0.003 which showed a high statistical significance as depicted in Table 4.

## Discussion:

It is of interest to assess the temperature differences during single drill and convention gradual and multiple drills procedures for placing dental implants. To assess the temperature difference, a thermal infrared camera was used. Using an infrared camera had many advantages over the thermocouple including an infrared camera assessing the temperature in a contactless manner and an infrared camera measuring the whole temperature, whereas, the thermocouple only measured a spot temperature as suggested by Frosch *et al*. [8] in 2019 and Markovic *et al*. [9] in 2013. The present study assessed this alteration in temperature on the artificial bone blocks which is also supported by the previous studies of Mohlhenrich *et al*. [10] in 2016 and Mohelhenrich *et al*. [11] in 2015 where similar artificial bone blocks were used by the authors in their studies to place the dental implants. These blocks have been widely accepted and a standardized tool for placing dental implants making them ideal to be used in the present study to assess different drilling procedures for osteotomy. The artificial bone blocks used were made of SRPF (solid rigid polyurethane foam) which were in the bone density of D1 which density of 0.48 g/cm3. These blocks were utilized owing to their various advantages including standard bone density, providing equal horizontal and vertical parameters, less error sensitivity, and good reproducibility. Frosch L in their study used the bovine ribs for drilling. However, bovine ribs had disadvantages including surface fluid retention masking actual readings, different mineralization grades, and different densities. A recent study by Bacci *et al*. [12] in 2020 used ex vivo human bone samples that lacked reproducibility and standardization owing to changes in the shape and density of the bone specimens. The present study used the artificial bone blocks of density d1 as the d1 bone type is considered to be the most vulnerable bone type to heat among all existing densities as also supported by the study of Mohelhenrich *et al*. [11] in 2015. After all the preparations were done for the present study, the initial temperature was denoted by T0 which was found to be in the range of 15.8-28.5°C. The maximum change in the temperature was denoted by ΔT which was caused by the drilling of the bone block to prepare the osteotomy sites, This was kept following the first law of thermodynamics which was also followed by Luccihiari *et al*. [12] in 2014 where authors reported that heat quantity absorbed by the bone block and the followed raise in the temperature is completely independent of the initial sample's temperature (T0). The thickness of the remaining wall of the artificial bone block following the completion of the drilling process was kept between 0.3mm to 0.7mm to allow the infrared camera to an accurate reading of the temperature change. This was contradictory to the findings of Matthews and Hirsch [13] in 1972 where authors reported that surface temperature assessed decreases proportionally with increased distance between the surface and drilling site. In the present study, the drilling in the artificial bone blocks was done at a speed of 1500 rpm which was consistent with the pioneer study of 1986 by Eriksson and Adell [14] However, other studies of Tehemar [15] in 1999 reported that lesser heat is generated by drilling at a lower speed. However, drill speed cannot be considered as an individual predictor for heat generation during drilling. The load applied during the drilling process in the present study was entirely based on the choice of the operator where they were allowed to adjust the load depending on the resistance in bone felt during the drilling process. However, as suggested by Misch, [16] drilling pressure should not be as high to increase heat and decreased cutting efficiency, and should not be light which only leads to heat generation without bone cutting. During the drilling process of the present study, continuous irrigation was done with the normal saline at room temperature at the rate of 50 ml/min. This was following the studies of Rashad *et al*. [17] in 2011 and Sener *et al*. [18] in 2009 where authors reported that saline irrigation at room temperature provides adequate cooling during the bone drill, however, higher irrigation volume does not decrease the heat generated during the drilling process. Koutiech S *et al*. [19] discussed similar results stating that the turning speed of the drill itself cannot be considered as an independent factor in heat generation during drilling without associating it with the load applied during drilling. Despite using identical bone blocks, the temperature was different which can be attributed to irrigant accessibility to the bone, speed, and tolerance amount of the bone. The study had a few limitations including the in-vitro nature which might not exactly simulate the intraoral conditions of the mouth, the temperature was not similar to the oral temperature, but was at room temperature, and blood flow was also not seen in the artificial bone blocks which are associated with human alveolar bone. Hence, the temperature change in the present study cannot be considered identical to the temperature alteration which could be seen in the oral cavity. Micro cracks following heat generation could also not be assessed in the present study.

## Conclusion:

Data shows that a single drill protocol for preparing an osteotomy site for placing a dental implant of diameter 4.2 generates lesser heat than conventional gradual drilling protocols.

## Figures and Tables

**Table 1:** Initial (T0) and maximum (Tmax) temperature during osteotomy preparation

**Conventional drills**					**Single drill 4.0mm (T0-Tmax)**
**2.0 mm (T0-Tmax)**	**2.8 mm (T0-Tmax)**	**3.0 mm (T0-Tmax)**	**3.5 mm (T0-Tmax)**	**4.0 mm (T0-Tmax)**	
23.4-26.7	23.1-34.2	24.8-29.2	23.8-24.6	23.3-24.0	22.1-30.0
23.1-25.9	22.8-28.1	24.8-31.2	22.8-24.6	23.3-26.4	21.8-26.0
24.4-29.2	24.8-34.1	23.3-24.4	23.8-25.6	24.3-25.4	22.8-24.6
25.3-27.9	23.3-31.1	23.3-24.0	24.3-26.0	24.3-27.1	21.8-29.7
25.3-27.7	24.8-28.8	25.3-28.5	24.3-25.7	24.3-25.9	22.8-27.5
25.3-31.9	24.8-37.9	25.3-28.8	24.8-25.9	24.3-27.0	22.3-33.9
28.3-33.0	25.8-35.8	26.1-32.1	24.8-28.3	24.3-25.7	22.3-23.8
28.3-32.1	15.8-30.6	26.1-29.2	26.0-33.8	24.8-26.7	22.3-32.8
27.1-29.8	26.1-44.8	26.1-28.6	26.0-33.9	24.8-26.0	28.3-31.3
27.1-29.8	26.1-34.1	26.1-30.2	27.2-36.1	26.8-36.1	28.3-30.0
26.8-32.6	26.1-37.0	26.131.5	28.5-33.6	26.8-32.6	28.0-31.1
26.1-29.1	25.8-36.3	25.8-31.6	26.8-32.6	26.8-32.9	28.0-32.1
26.4-28.8	25.7-36.4	25.6-31.4	26.6-32.4	26.6-32.7	28.0-32.1

**Table 2:** Maximum recorded temperature alterations in two groups of study during osteotomy

**Conventional drills**					**Single drill 4.0mm (ΔT)**
**2.0mm (ΔT)**	**2.8mm (ΔT)**	**3.0mm (ΔT)**	**3.5mm (ΔT)**	**4.0mm (ΔT)**	
3.1	0.9	4.2	0.6	0.5	7.7
2.6	5.1	6.2	1.6	2.9	4
4.8	9.1	0.9	1.6	0.9	1.6
2.4	7.6	0.5	1.5	2.6	7.7
2.2	3.8	3	1.2	1.4	4.5
6.4	2.9	3.3	0.9	2.5	11.4
4.5	9.8	5.8	3.3	1.2	1.3
3.6	4.6	2.9	7.6	1.7	10.3
2.5	18.8	2.3	7.7	1	2.8
2.5	7.8	3.9	8.7	9.1	1.5
5.6	10.7	5.2	4.9	5.6	2.9
2.8	10.3	5.6	4.6	5.9	3.9
2.6	10.1	5.4	4.4	5.7	3.7

**Table 3:** Maximum and minimum limit of temperature change in study groups

**Preparation Method**	**Number (n)**	**Mean± S.D**	**ΔT max limit**	**ΔT min limit**
Group I	13	5.15±3.45	1.3	11.4
Group II	13	9.89±3.64	3.8	18.5

**Table 4:** Temperature change difference between the study groups

**Parameter**	**Mean difference**	**T**	**p-value**
ΔT	-4.72	-3.254	0.003
